# Immune-Mitochondrial Crosstalk in Pancreatic Adenocarcinoma: Systematic Identification of Prognostic Biomarkers Through Immune Dictionary Framework

**DOI:** 10.2174/0118715303422686250925054821

**Published:** 2025-10-15

**Authors:** Jiangang Zhao, Fenglin Zhang, Xinghe Liao, Ke Zhang, Ping Li, Hao Chen

**Affiliations:** 1 Department of Oncology, Shaoxing Central Hospital, Shaoxing, 312030, China;; 2 Department of Oncology, The Central Affiliated Hospital, Shaoxing University, Shaoxing, 312030, China;; 3 Oncology Department of Integrated Traditional Chinese and Western Medicine, The First Affiliated Hospital of Anhui Medical University, Hefei, 230022, China;; 4 Graduate School of Anhui University of Traditional Chinese Medicine, Hefei, 230022, China;; 5 Department of Integrative Oncology, Fudan University Shanghai Cancer Center, Shanghai, 200032, China;; 6 Department of Oncology, Shanghai Medical College, Fudan University, Shanghai, 200032, China

**Keywords:** Pancreatic adenocarcinoma, immune dictionary, mitochondrial dysfunction, EGF/EGFR, bioinformatics, prognostic model

## Abstract

**Introduction:**

Pancreatic adenocarcinoma (PAAD) is characterized by aggressive progression, driven by an immunosuppressive tumor microenvironment (TME) and mitochondrial dysfunction. Alterations in mitochondrial bioenergetics and metabolic reprogramming are crucial to tumor survival, invasion, and immune evasion. The “immune dictionary” approach provides a systematic classification of immune-related genes, offering insights into immune dysfunction and its interaction with mitochondrial pathways in the context of PAAD outcomes.

**Methods:**

To identify differentially expressed genes (DEGs) associated with immune dysfunction and mitochondrial pathways in PAAD, data from TCGA, GTEx, and three GEO datasets were integrated. Differential gene expression was analyzed using DESeq2, applying criteria of *p*-value < 0.05 and |log_2_ fold change| > 1. Using the “immune dictionary” framework, a prognostic model was developed through LASSO-Cox regression, followed by survival analysis for validation. The expression of key genes identified in the bioinformatics analysis was validated by quantitative real-time PCR (qPCR) on paired PAAD tissue and adjacent normal samples, focusing on NOG, TNFSF9, and TNFSF10.

**Results:**

Fifty-two DEGs associated with immune and mitochondrial dysfunction were identified. Gene set enrichment analysis revealed critical pathways, including IL-4 signaling, NF-κB activation, and autophagy. The LASSO-Cox model identified 12 prognostic genes that effectively stratified patients into high- and low-risk groups with high predictive accuracy. Validation confirmed significant differential expression patterns, consistent with computational findings.

**Discussion:**

This study, utilizing the immune dictionary framework, systematically analyzed immune-related and mitochondria-related genes in PAAD, identifying 12 DEGs for prognostic modeling. It revealed significant correlations between immune evasion and mitochondrial dysfunction, offering novel targets for personalized therapy.

**Conclusion:**

This study presents an innovative immune dictionary approach to identify key immune- and mitochondria-related DEGs in PAAD, providing potential targets for new therapeutic strategies and personalized treatment approaches.

## 1 INTRODUCTION

With a global five-year survival rate of only 13%, pancreatic adenocarcinoma (PAAD) ranks among the most aggressive and lethal cancers [1, 2]. In 2020, PAAD was responsible for 466,003 deaths worldwide, and its incidence is expected to increase [3]. By 2030, it is projected to become the second leading cause of cancer-related mortality [1, 3]. The poor prognosis is primarily attributed to its silent progression during the early stages, resulting in diagnoses at a point when surgical intervention is often no longer viable [4-6]. Current chemotherapy regimens, such as FOLFIRINOX and gemcitabine, provide only modest survival benefits, particularly for patients in advanced stages [7-9]. Tumor resistance to conventional therapies, coupled with the immunosuppressive tumor microenvironment (TME), highlights the pressing need for innovative therapeutic approaches [10, 11].

The immunosuppressive TME of PAAD presents a major hurdle to effective treatment. This microenvironment is characterized by a dense fibrotic stroma, limited infiltration of effector immune cells, and an elevated presence of regulatory T cells (Tregs) and tumor-associated macrophages (TAMs), all of which hinder antitumor immune responses [12, 13]. While immunotherapy has demonstrated significant success in treating cancers like melanoma and lung cancer, its effectiveness in PAAD remains constrained due to the suppressive TME and robust immune evasion mechanisms [14, 15]. Additionally, recent research highlights the role of metabolic reprogramming, particularly mitochondrial dysfunction, in promoting tumor growth and supporting immune evasion within the TME [16-18].

Mitochondria play a pivotal role in regulating cellular metabolism and immune responses. In cancer, mitochondrial dysfunction is linked to impaired immune function, limiting the metabolic capacity of key immune cell populations such as CD8+ T cells and natural killer (NK) cells, leading to immune exhaustion and diminished antitumor activity [19-21]. Despite growing insights into the connection between mitochondrial dysfunction and immune regulation, the specific interactions within PAAD remain poorly understood.

To tackle these challenges, this study introduces the concept of an “immune dictionary”, a structured framework for systematically cataloging immune-related genes and their interactions within the TME of PAAD [22]. This research aims to explore the relationship between immune suppression, mitochondrial dysfunction, and PAAD progression using this novel approach. By establishing this immunological dictionary, this study aims to enhance understanding of the molecular pathways driving PAAD and identify new therapeutic targets, ultimately advancing treatment strategies.

## 2 MATERIALS AND METHODS

### 2.1 Data Download

The PAAD dataset, which includes sequencing data from 178 pancreatic cancer cases and 4 control specimens, was retrieved from The Cancer Genome Atlas (TCGA) using the TCGA biolinks R package [23] (Version 2.30.0). Due to the limited availability of control samples, TCGA data were merged with pancreatic data from the Genotype-Tissue Expression (GTEx) project, which was normalized to FPKM. Clinical information was obtained from UCSC Xena [24]. The GEOquery R package [25] (version 2.70.0) was used to retrieve the PAAD datasets GSE15471 [26], GSE28735 [27], and GSE62452 [28] from the Gene Expression Omnibus (GEO) database [26, 29]. Mitochondrial-related genes (MRGs) were sourced from GeneCards [30], resulting in a total of 12,344 MRGs. Additionally, the Dictionary of Immune Response-Related Genes (DIRRGs) was compiled by searching the PubMed database (https://pubmed.ncbi.nlm.nih.gov/) using the keyword “Dictionary of Immune Responses.” Core immune-related genes were extracted from the retrieved literature, primarily from the publication cited in a previous study [22], which provided a representative set of DIRRGs. Only genes involved in key immune pathways, such as innate immunity, adaptive immune responses, antigen presentation, cytokine secretion, and T/B cell activation, and supported by experimental validation or inclusion in well-established immune-related pathways, were retained. After screening and removing duplicates, a total of 86 DIRRGs were finalized for subsequent analysis (Table **1**).

### 2.2 Immune Dictionary Related to Pancreatic Cancer & Mitochondrial-related Differentially Expressed Genes

The TCGA-GTEx-PAAD dataset was stratified into control and PAAD cohorts. Differential expression analysis was conducted using DESeq2 [31], with a threshold of |logFC| > 0 and *p*-value < 0.05. Genes that were either upregulated or downregulated were visualized using ggplot2 (version 3.4.4). Pancreatic cancer-specific differentially expressed, mitochondrial-related genes (DIR&MRDEGs) were identified by intersecting DEGs with DIRRGs and MRGs and were visualized using a Venn diagram.

### 2.3 Gene Set Enrichment Analysis (GSEA)

GSEA [32] was performed on the TCGA-GTEx-PAAD dataset using the clusterProfiler R package [33] (version 4.10.0), with genes ranked by logFC. Canonical pathways from the MSigDB database [34] were utilized, and *p*-values were used to determine significance. The parameters were set to seed = 2020, 1000 permutations, with a minimum of 10 genes and a maximum of 500 genes per gene set.

### 2.4 Somatic Mutation (SM) and Copy Number Variation (CNV) Analysis

SM in pancreatic cancer samples from the TCGA-GTEx-PAAD dataset were analyzed using the 'Masked Somatic Mutation' data from TCGA, processed with VarScan software. The maftools R package [35] (version 2.18.0) was employed to visualize these mutations. For CNV analysis, the 'Masked Copy Number Segment' dataset from TCGA was used, and the types and frequencies of CNVs were visualized using ggplot2 (version 3.4.4).

### 2.5 Construction of Prognostic Model

To construct a prognostic risk model using the TCGA-GTEx-PAAD dataset, simple and complex Cox regression analyses were performed with the survival R package [36] (version 3.5-7). The objective was to investigate how immune dictionary- and mitochondria-associated differentially expressed genes (DIR&MRDEGs) influence survival outcomes. Variables with a *p*-value less than 0.10 in the univariate analysis were selected for LASSO regression. The glmnet package [37] (version 4.1-8) was used, with 'deviance' as the tuning parameter, and 10 iterations were performed. LASSO regression enhances model generalizability by incorporating a penalty term to reduce overfitting. A prognostic risk model and a variable trajectory diagram were generated to present the data, and genes suitable for module development were identified.

Multivariate Cox regression analysis was performed using the LASSO risk score and clinical data to calculate the risk score using the following formula (Eq.1):

*Score* = ∑_i_ Coefficient (gene_i_)*mRNA Expression (gene_i_) (1)

A forest plot was used to visualize the results of the multivariate Cox regression, presenting the LASSO risk score alongside clinical variables. Additionally, a nomogram was constructed using the rms R package [38] (Version 6.7-1) to illustrate the relationship between the LASSO risk score and clinical parameters.

High-risk and low-risk groups were defined based on the median RiskScore by combining the expression levels of module genes with their corresponding LASSO coefficients, thereby validating the prognostic risk model. Kaplan-Meier (KM) survival analysis [39] was conducted using the survival R package to compare overall survival (OS) between these groups. Model performance was assessed using time-dependent Receiver Operating Characteristic (ROC) curves, with AUC values indicating accuracy. AUC values greater than 0.5 indicate a positive correlation, values between 0.5 and 0.7 suggest low accuracy, values between 0.7 and 0.9 reflect moderate accuracy, and values above 0.9 represent high accuracy. ROC curves, predicting 1-, 3-, and 5-year OS, were generated using the survival ROC package (Version 1.0.3.1) [40].

The model’s predictive accuracy was evaluated through a calibration curve comparing actual and expected probabilities. Decision curve analysis (DCA) using ggDCA (version 1.1) [41] further assessed the clinical utility of the model.

Using the TCGA-GTEx-PAAD and GEO datasets (GSE15471, GSE28735, and GSE62452), comparison maps were generated to analyze modulator gene expression variations between the control and PAAD groups. A heatmap was created using the Pheatmap package (version 1.0.12). Correlation and chord diagrams were constructed with Igraph (Version 1.6.0) and ggraph (Version 2.1.0) to visualize the data. Spearman’s correlation was applied to examine the relationships between module genes, with correlation strength categorized as follows: little or no correlation (|r| < 0.3), weak correlation (0.3-0.5), moderate correlation (0.5-0.8), and strong correlation (> 0.8).

### 2.6 Gene Ontology (GO) and Kyoto Encyclopedia of Genes and Genomes (KEGG) Enrichment Analyses

Biological processes (BP), molecular functions (MF), and cellular components (CC) are integral categories within GO [42], which is employed in functional enrichment analysis. KEGG [33], an extensive resource integrating data on metabolic pathways, medicinal drugs, disease processes, and genomic sequences, was also utilized. GO and KEGG enrichment analyses of module genes were performed using the clusterProfiler R package [43]. *P*-values were corrected using the Benjamini-Hochberg (BH) method to ensure statistical significance, with a false discovery rate (FDR, q-value) of less than 0.05.

### 2.7 Gene Set Variation Analysis (GSVA)

GSVA [44], a non-parametric, unsupervised method, was applied to assess gene set enrichment. This approach transforms the gene expression matrix across different specimens to determine if certain biological pathways are over- or underrepresented in specific datasets. For GSVA on the extensive TCGA-GTEx-PAAD dataset, the h.all.v7.4.symbols.gmt gene set from the Molecular Signatures Database (MSigDB) was used. *P*-values were adjusted using the BH method to ensure that the GSVA screening criterion was less than 0.05.

### 2.8 Construction of Pancreatic Cancer Subtypes

Consensus Clustering, a resampling-based algorithm [45], was employed to identify subgroups and evaluate clustering stability within pancreatic cancer samples from the TCGA-GTEx-PAAD dataset using the ConsensusClusterPlus R package [46] (Version 1.62.0). The number of clusters was set between two and nine, with 80% of the samples resampled 50 times (clusterAlg = 'km,’ distance = 'spearman'). Differences in module gene expression across subtypes were visualized using heatmaps and further validated with group comparison maps. Correlations between module genes were illustrated using a heatmap generated by the pheatmap package (version 1.0.12).

### 2.9 Analysis of Immunotherapy and Immune Infiltration

Boxplots were used to analyze variations in RiskScore across patients with different immune responses in the gastric cancer (GC) immunotherapy dataset (Kim 2018) [47]. The relative abundance of immune cell infiltrates, including activated CD8+ T cells, active dendritic cells, NK cells, and Tregs, was measured using Single-Sample GSEA (ssGSEA) [48]. An immune cell infiltration matrix was created using the enrichment scores obtained from ssGSEA, which indicate the relative proportions of these cells in the TCGA-GTEx-PAAD dataset. Comparative group maps showing immune cell expression across the high- and low-risk score groups were created using ggplot2 (Version 3.4.4). Significant immune cell populations were selected for further analysis. Spearman correlation was used to examine the relationships between immune cells, with the results presented in correlation heatmaps generated using the pheatmap package (Version 1.0.12). Additionally, correlations between module genes and immune cells were assessed using Spearman’s method (*p* < 0.05) and visualized with correlation bubble plots created using ggplot2.

### 2.10 qRT-PCR

RNAex (1 mL; Agbio, AG21101, China) was added to the cells. After thorough mixing and centrifugation, the upper liquid phase was discarded. The total RNA precipitate was then purified with a 75% ethanol solution, followed by reverse transcription to yield RNA. The detailed protocol was carried out according to the EasyScript First-Strand cDNA Synthesis SuperMix (TransGen, AE301, China) and ChamQ Blue Universal SYBR qPCR Master Mix (Vazyme, AG21101, China) guidelines. The PCR amplification program used an annealing temperature of 95°C, with 40 cycles. Real-time PCR analysis was performed using the ABI PRISM® 7500 Sequence Detection System (Applied Biosystems, USA) following the operational protocols. Gene expression at the mRNA level was quantified using the 2−ΔΔCt method, with final values normalized to β-actin expression. The primer sequences for NOG were: CATCCAGTACCCCATCATTT (F), ATTGAAAACCCTCGCTAGAG (R); TNFSF10 primers: GTGAAACCCCATCTCTACTGA (F), GAGTGTAGTGGCATGATCTCA (R); TNFSF9 primers: TGCTGCTTTCTCTACCTCAA (F), CCCCAAAACAACTCCTCTAA (R); GAPDH primers: GGGAAACTGTGGCGTGAT (F), GAGTGGGTGTCGCTGTTGA (R).

### 2.11 Statistical Analysis

Data analysis was performed using R software (Version 4.2.2). Means and standard deviations (SD) of continuous variables were reported. The Kruskal-Wallis test was used for comparisons involving three or more groups, while the Wilcoxon rank-sum test was applied for comparisons between two groups. Fisher’s exact test or the chi-square test was used to evaluate categorical variables. Statistical significance was set at a *p*-value of less than 0.05. Spearman correlation analysis was used unless otherwise specified.

## 3 RESULTS

### 3.1 Technology Roadmap

**Fig. (1):**
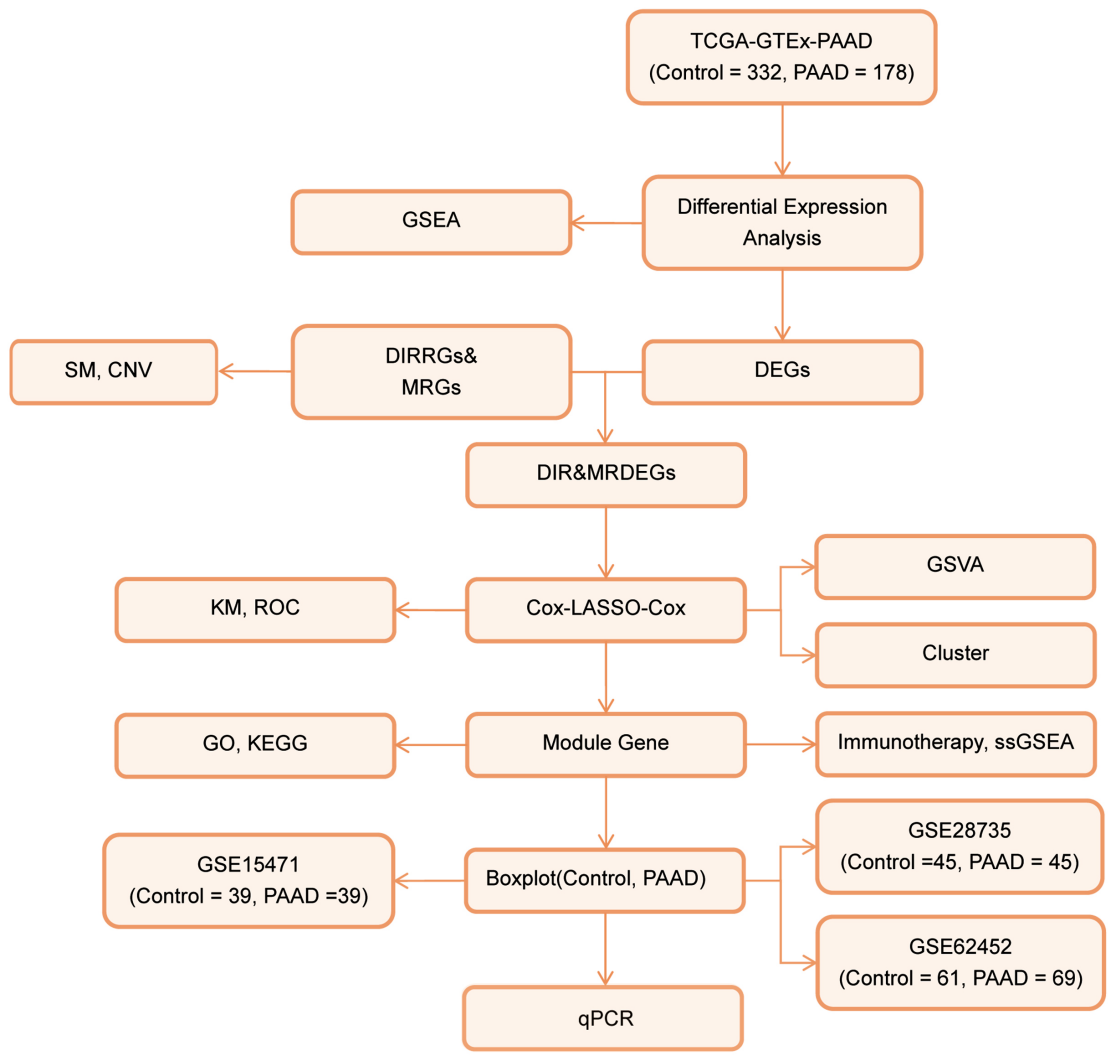
Technology roadmap.

Adenocarcinoma of the pancreas (PAAD) and The Cancer Genome Atlas (TCGA) genes exhibit varying expression patterns. Metabolism-Related Genes (MRGs), “DIRRGs” (Dictionary of Immune Responses Related Genes), and “DIR&MRDEGs” (Dictionary of Immune Responses & Mitochondrial Related Differentially Expressed Genes) were analyzed in this study. Other key terms include Somatic Mutation (SM), Copy Number Variations (CNV), Gene Set Enrichment Analysis (GSEA), Gene Set Variation Analysis (GSVA), Receiver Operating Characteristic (ROC), Least Absolute Shrinkage and Selection Operator (LASSO), Gene Ontology (GO), Kaplan-Meier (KM), and Kyoto Encyclopedia of Genes and Genomes (KEGG). Additionally, Single-Sample Gene Set Enrichment Analysis (ssGSEA) was employed for assessing immune cell infiltrates. Furthermore, qPCR analysis was performed on paired PAAD tissue samples to confirm the expression levels of key genes identified through computational approaches (Fig. **1**).

### 3.2 Immune Dictionary Related to Pancreatic Cancer & Mitochondrial-related Differentially Expressed Genes

**Fig. (2):**
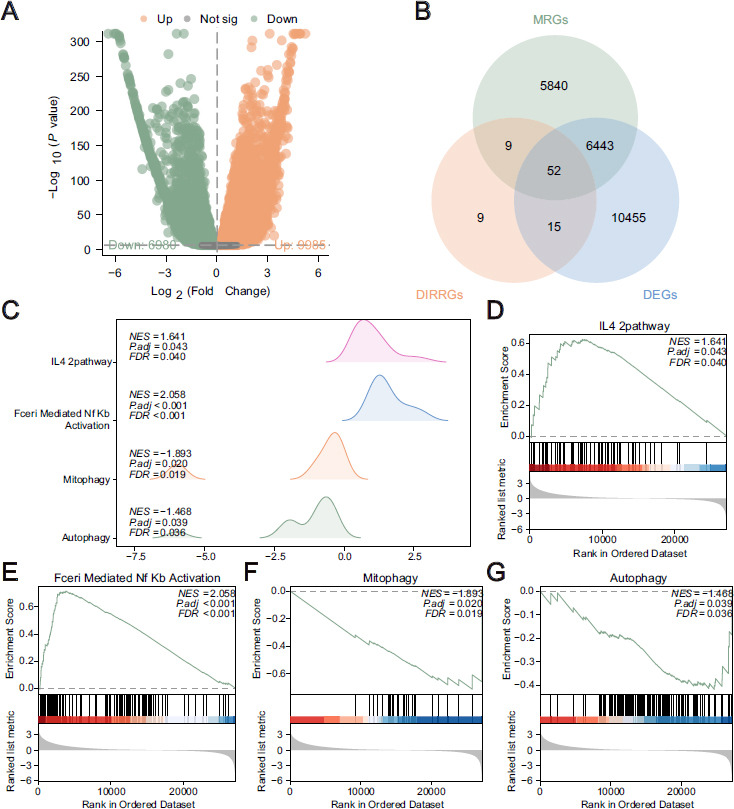
Gene Set Enrichment Analysis (GSEA). (**A**) Volcano plot of differentially expressed genes (DEGs) between PAAD and controls in the TCGA-GTEx-PAAD dataset. Red and blue dots represent significantly upregulated and downregulated genes (*p* < 0.05), respectively. (**B**) Venn diagram depicting the intersection of DEGs (16,965), immune-related genes (DIRRGs, 86), and mitochondrial-related genes (MRGs, 12,344), identifying 52 DIR&MRDEGs relevant to PAAD. (**C**) GSEA plot showing pathway enrichment distribution based on gene ranking. (**D-G**) Significantly enriched pathways from GSEA analysis: (**D**) IL-4 signaling (PID_IL4_2PATHWAY): NES = 1.64, *p*.adj = 0.0429, q = 0.0405; (**E**) FcεRI-mediated NF-κB activation (REACTOME_FCERI_MEDIATED_NF_KB_ACTIVATION): NES = 2.06, *p*.adj = 1.39 × 10^–8^, q = 1.31 × 10^–8^; (**F**) Mitophagy (REACTOME_MITOPHAGY): NES = -1.89, *p*.adj = 0.0205, q = 0.0193; (**G**) Autophagy (REACTOME_AUTOPHAGY): NES = -1.47, *p*.adj = 0.0385, q = 0.0363. Significance was determined by adjusted *p*-value < 0.05 and FDR < 0.05 (Benjamini-Hochberg correction).

To investigate potential variations in gene expression between the control and PAAD groups in the TCGA-GTEx-PAAD dataset, differential expression analysis was conducted using DESeq2. This analysis identified 16,965 DEGs with thresholds of |logFC| > 0 and *p*-value < 0.05. Among these, 6,980 genes were upregulated, while 9,985 were downregulated (Fig. **2A**).

To further refine the selection of genes relevant to immune responses and mitochondrial function, an intersection analysis was performed. By cross-referencing DIRRGs and MRGs, 52 DEGs (DIR&MRDEGs) associated with both immune and mitochondrial functions were identified. Additionally, 61 genes related to both the immune dictionary and mitochondrial function (DIR&MRGs) were identified. These results are visually represented in a Venn diagram (Fig. **2B**) and listed in Table **S1**.

### 3.3 GSEA

The TCGA-GTEx-PAAD dataset was also utilized to assess the effect of gene expression on adenomas. This analysis aimed to explore the relationships between gene expression profiles and the functions of BP, CC, and MF (Fig. **2C**). Table **2** summarizes the comprehensive outcomes of this analysis. Significant enrichment of genes across several crucial pathways was observed, including the IL-4 signaling pathway (Fig. **2D**), FcεRI-mediated NF-κB activation (Fig. **2E**), mitophagy (Fig. **2F**), autophagy (Fig. **2G**), and IL-6/STAT3 signaling pathway, as well as other critical biological processes and signaling pathways.

### 3.4 Immune Dictionary & SM and CNV of MRGs

**Fig. (3):**
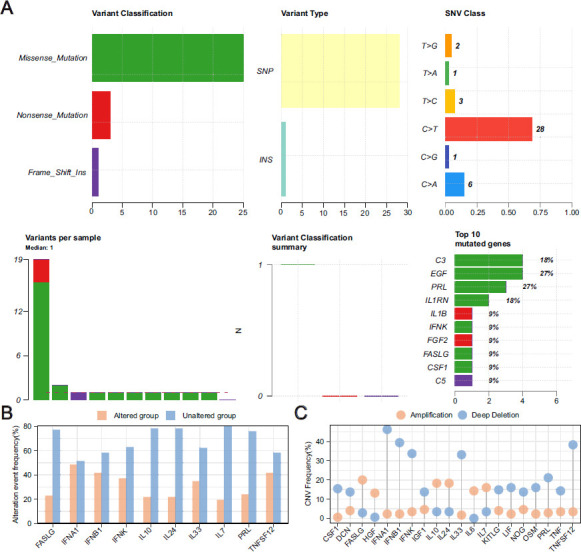
Somatic Mutation (SM) and Copy Number Variation (CNV) Analysis. (**A**). Somatic mutation (SM) profile of Dictionary of Immune Responses & Mitochondrial-related genes (DIR&MRGs) in pancreatic cancer. (**B-C**). Copy number variation (CNV) analysis of DIR&MRGs in pancreatic cancer, showing mutation frequency bar chart (**B**) and mutation type lollipop plot (**C**).

The SM in the 61 DIR&MRGs in pancreatic cancer were examined by compiling mutation data and visualizing it using the maftools R program (Fig. **3A**). The results revealed that missense mutations were the most common among the three main mutation types. Single-nucleotide polymorphisms (SNPs) have recently become the predominant mutation type, with C-to-T transitions being the most frequent single-nucleotide variation (SNV).

For CNV analysis of the same set of 61 DIR&MRGs, pancreatic cancer CNV data were combined and processed using GISTIC2.0. The analysis showed that all 61 genes exhibited CNVs, with the top 10 and 20 genes displaying the most frequent variations (Figs. **3B** and **C**). The percentages represent the frequency distribution of CNVs in the 61 DIR&MRGs across samples, detailing the specific occurrence rates for each gene without applying statistical thresholds to define “significant” CNV alterations. Among the genes exhibiting significant CNV alterations were CSF1, DCN, FASLG, HGF, IFNA1, IFNB1, IFNK, IGF1, IL10, IL24, IL33, IL6, IL7, KITLG, LIF, NOG, OSM, PRL, TNF, and TNFSF12.

### 3.5 Construction and Validation of a Prognostic Model for Pancreatic Cancer

**Fig. (4):**
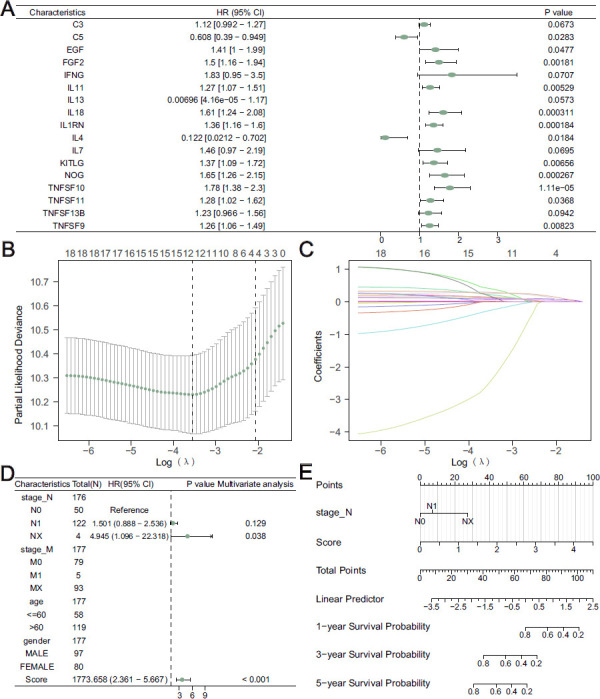
LASSO and cox prognostic analysis. (**A**) Forest plot of Cox univariate regression analysis results. (**B-C**) Plots of prognostic risk models (**B**) and variable trajectories (**C**) from LASSO regression. (**D-E**) Clinical data and LASSO risk score in a multivariate Cox regression model, accompanied by a forest plot (**D**) and nomogram (**E**). Acronyms used include Least Absolute Shrinkage and Selection Operator (LASSO) and Dictionary of Immune Responses and Mitochondrial Related Differentially Expressed Genes (DIR&MRDEGs).

A univariate Cox regression analysis was conducted to build a predictive risk model for pancreatic cancer using the TCGA-GTEx-PAAD dataset. To assess the predictive value of differentially expressed immune and mitochondrial-related genes (DIR&MRDEGs), clinical data were combined with these genes. The findings from the univariate Cox regression were visualized using a forest plot (Fig. **4A**), and variables with a *p*-value less than 0.10 were included in the LASSO regression analysis. To refine the prognostic model, 12 module genes —EGF, FGF2, IFNG, IL11, IL13, IL18, IL1RN, IL27, IL4, NOG, TNFSF10, and TNFSF9 —were selected, and LASSO regression was applied (Figs. **4B** and **C**). Subsequently, multivariate Cox regression was performed, incorporating the LASSO risk score (score) along with clinical data to evaluate the model’s prognostic performance. The results are presented using a forest plot (Fig. **4D**) and a nomogram illustrating the correlation between the score and clinical variables (Fig. **4E**). The LASSO risk score showed superior prognostic accuracy compared to other factors, with Stage_N demonstrating a relatively lower predictive value.

**Fig. (5):**
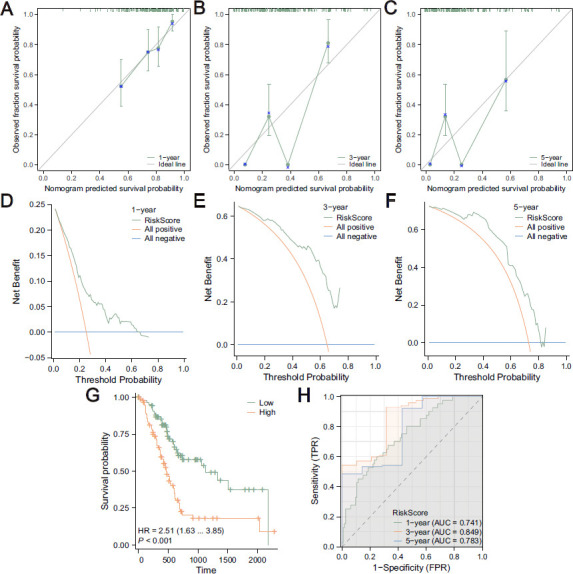
Prognostic analysis. **(A-C**) Calibration curves of the pancreatic cancer prognostic risk model at 1, 3, and 5 years, respectively. (**D-F**) Decision curve analysis (DCA) plot for the pancreatic cancer prognostic risk model at 1 year (**D**), 3 years (**E**), and 5 years (**F**). (**G**) presents a Kaplan-Meier (KM) curve illustrating the relationship between overall survival (OS) of patients with pancreatic cancer and their RiskScore. (**H**) Time-dependent ROC curve for patients with pancreatic cancer in the TCGA-GTEx-PAAD dataset. The acronyms TCGA, PAAD, OS, KM, ROC, AUC, DCA, TPR, and FPR refer to the “True Positive Rate” and “False Positive Rate,” respectively, in the context of studies such as the Cancer Genome Atlas and the Pancreatic Adenocarcinoma Database. This indicates a highly significant statistical relationship, as the *p*-value is less than 0.001. Diagnostic efficacy improves as the area under the curve (AUC) approaches 1, with a value greater than 0.5 suggesting a trend toward promoting the event. Accuracy is achieved when the AUC falls between 0.7 and 0.9.

Calibration curves were used to evaluate the model's accuracy at 1-, 3-, and 5-year intervals (Figs. **5A**-**C**). The results showed that the predicted and actual survival rates were closely aligned, indicating superior model performance.

DCA was performed for 1-, 3-, and 5-year predictions (Figs. **5D**-**F**), demonstrating that the model had the best clinical prediction capability at the 5-year mark compared to the 1-year prediction.

"Risk*Score* = Stage_NN1 stage_NNX * (0.40609) + Score (1.59841) * (1.29679) + (3.59972)

KM analysis (Fig. **5G**) was conducted to assess OS based on risk score stratification within the TCGA-GTEx-PAAD dataset. The analysis revealed a highly significant difference in OS between the high- and low-risk groups (*P* < 0.001). Furthermore, time-dependent ROC curve analysis (Fig. **5H**) demonstrated that the prognostic model had moderate predictive accuracy, with AUC values ranging from 0.7 to 0.9 for 1-, 3-, and 5-year predictions.

**Fig. (6):**
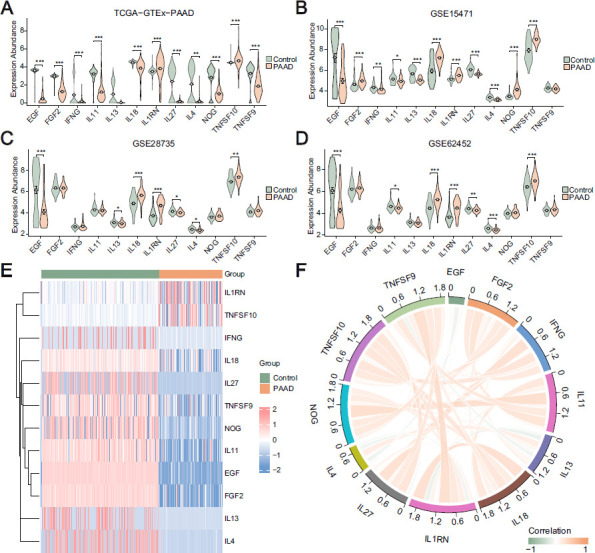
Expression analysis of module genes. **(A-D**) Group comparison of module genes between the PAAD and control groups in the TCGA-GTEx-PAAD dataset (**A**) and datasets GSE15471 (**B**), GSE28735 (**C**), and GSE62452 (**D**). (**E**) Heatmap of model genes (module genes) in the TCGA-GTEx-PAAD dataset. (**F**) Correlation and chord diagram of 11 model genes in the TCGA-GTEx-PAAD dataset. TCGA, The Cancer Genome Atlas; PAAD, Pancreatic Adenocarcinoma. ns, *p*-value ≥ 0.05, no statistical significance; *p-value < 0.05, **p-value < 0.01, ***p-value < 0.001. In the heatmap, green represents the control group, and orange represents the PAAD group, with blue indicating low expression and pink indicating high expression. A correlation coefficient (r-value) with an absolute value less than 0.3 is considered weak or insignificant, between 0.3 and 0.5 indicates moderate correlation, and above 0.8 indicates strong correlation. Positive correlations are shown by orange, and negative correlations by green.

Subsequently, the expression levels of the 12 module genes (EGF, FGF2, IFNG, IL11, IL13, IL18, IL1RN, IL27, IL4, NOG, TNFSF10, and TNFSF9) were examined across the TCGA-GTEx-PAAD and GEO datasets (GSE15471, GSE28735, and GSE62452). Group comparison plots (Fig. **6A**-**D**) revealed significant differential expression (*p* < 0.01) for 11 genes in the TCGA-GTEx-PAAD dataset. In GSE15471, 11 genes exhibited significant differences (*p* < 0.05), whereas seven genes (EGF, IL13, IL18, IL1RN, IL27, IL4, and TNFSF10) showed significant differences in expression in GSE28735. In GSE62452, seven genes exhibited significant differential expression (*p* < 0.05).

Additionally, to observe variations in gene expression of the module genes among different TCGA-GTEx-PAAD sample groups, a heatmap was generated using the pheatmap R package (Fig. **6E**). Correlation and chord diagrams (Fig. **6F**) were constructed to assess the relationships between the 12 module genes, revealing predominantly positive correlations.

### 3.6 GO and KEGG Enrichment Analyses

Table **3** presents the comprehensive results of the GO and KEGG pathway enrichment analyses conducted using the 12 module genes. The GeneRatio reflects the proportion of module genes enriched in a specific pathway relative to the total number of module genes. These findings indicate that the genes are primarily involved in key BP, such as regulation of ATP metabolism, DNA metabolic regulation, cell growth control, B cell activation, and NK T cell activation. In terms of MF, the genes are enriched in activities related to cytokines, receptor-ligand interactions, growth factors, signaling receptor activators, and interleukin-1 receptor binding. KEGG pathway analysis revealed enrichment in several crucial signaling pathways, including the JAK-STAT, IL-17, T cell receptor, HIF-1, and PI3K-Akt signaling pathways.

**Fig. (7):**
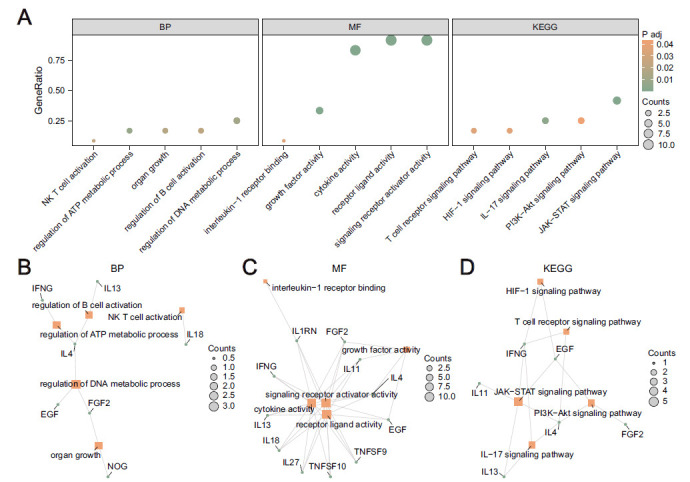
Gene ontology (GO) and pathway (KEGG) enrichment analysis. **(A**) Analysis of 12 model genes (module genes) for biological process (BP), molecular function (MF), and biological pathway (KEGG) and Gene Ontology (GO) enrichment (bubble diagram). The GO and KEGG terms are represented on the x-axis. The network diagram analysis highlights BP (**B**), MF (**C**), and KEGG (**D**) pathways for the model genes (module genes) in GO and KEGG. Green nodes represent molecules, entry-representing attachments, and molecular relationships, while orange nodes represent entries. The acronyms for “Gene Ontology,” “Biological Process,” and “Molecular Function” are used, with KEGG referring to the Kyoto Encyclopedia of Genes and Genomes. In the bubble plot, the size of the bubble represents the number of genes. In contrast, the color of the bubble corresponds to the adjusted *p*-value (*p*.adj), with smaller colors indicating more orange (lower *p*.adj values) and larger colors indicating greener shades (higher *p*.adj values). GO and KEGG pathway enrichment analyses required a *p*-value < 0.05 and an FDR (q-value) < 0.05, with the BH correction method applied to the *p*-value.

To visualize the relationships between these enriched functions and pathways, a bubble chart (Fig. **7A**) was used, with larger bubbles representing a higher degree of gene involvement in specific processes or pathways.

Additionally, network diagrams (Figs. **7B**-**D**) were constructed to highlight the interconnections among biological processes, molecular functions, and signaling pathways, illustrating how these genes contribute to key regulatory mechanisms in pancreatic cancer.

### 3.7 GSVA

**Fig. (8):**
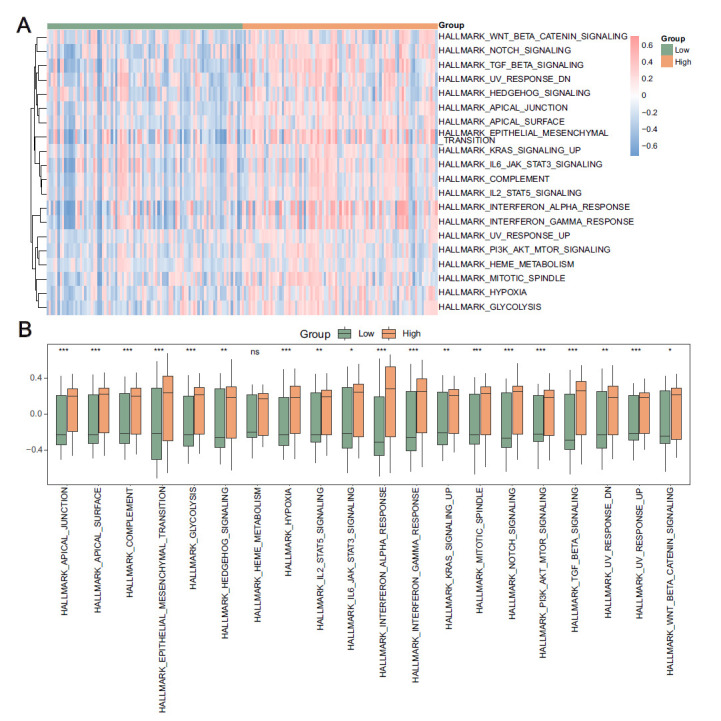
Gene set variation analysis (GSVA). (**A, B**) Group comparison map (**B**) and heatmap (**A**) comparing GSVA results between the High and Low groups of the TCGA-GTEx-PAAD dataset. The acronyms TCGA, PAAD, and GSVA represent “The Cancer Genome Atlas,” “Pancreatic Adenocarcinoma,” and “Gene Set Variation Analysis,” respectively. In the expression map, green indicates the low-risk group, orange represents the high-risk group, pink signifies high expression, and blue indicates low expression. The GSVA screening criteria were set to *p*.adj < 0.05, with the Benjamini-Hochberg (BH) method applied for *p*-value correction. Statistical significance is denoted as *p-value < 0.05, **p-value < 0.01, ***p-value < 0.001.

To explore variations in the h.all.v7.4.symbols.gmt gene set between the high-risk and low-risk groups in the TCGA-GTEx-PAAD cohort, GSVA was performed. The top ten positively and negatively enriched pathways (*p*.adj < 0.05) were selected for further analysis and visualized in a heatmap to highlight their differential expression (Fig. **8A**, Table **4**).

To confirm the significance of these differences, Mann-Whitney U tests were conducted, resulting in a group comparison map (Fig. **8B**). GSVA identified substantial differences in several pathways, including interferon alpha and gamma response, TGF-β signaling, epithelial-mesenchymal transition, Notch signaling, glycolysis, hypoxia, and mitotic spindles between the high- and low-risk groups (*p* < 0.05).

### 3.8 Construction of Pancreatic Cancer Subtypes

**Fig. (9):**
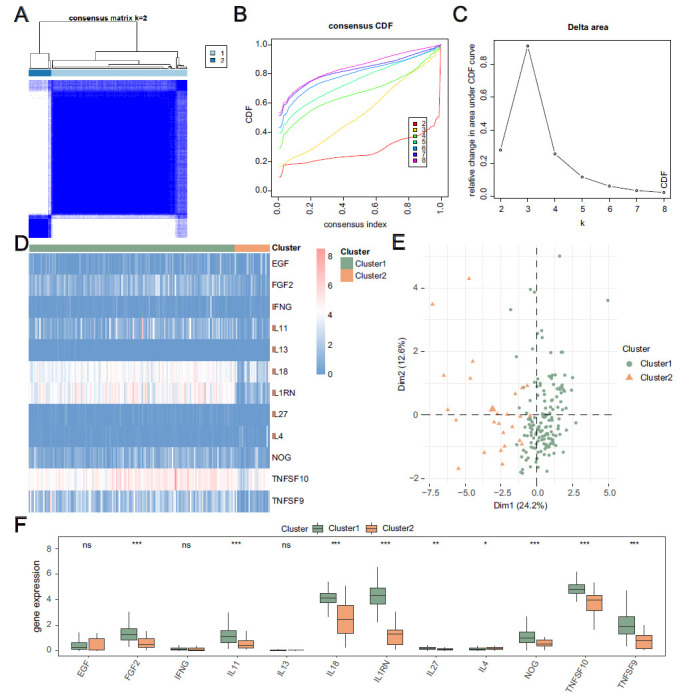
Construction of pancreatic cancer subtypes. (**A**) Plot of consensus clustering results for pancreatic cancer samples in the TCGA-GTEx-PAAD dataset. (**B-C**) Consistency cumulative distribution function (CDF) plot (**B**) and Delta plot (**C**) from consensus clustering analysis. (**D**) Heatmap of module gene expression values in pancreatic cancer subtypes. (**E**) PCA cluster map of two pancreatic cancer disease subtypes. (**F**) Cluster comparison of module genes between the two pancreatic cancer subtypes. Statistical significance is denoted as ns, *p* ≥ 0.05 (no statistical significance), *p < 0.05, **p < 0.01, ***p < 0.001. Green represents subtype A (Cluster 1), and orange represents subtype B (Cluster 2). In the heatmap, pink indicates upregulated expression, and blue indicates downregulated expression.

Using the expression levels of the 12 module genes, the ConsensusClusterPlus R program was employed to investigate disease subtypes in the TCGA-GTEx-PAAD pancreatic cancer dataset. Subtype A was identified in cluster 1, comprising 151 samples, while Subtype B was found in cluster 2, comprising 25 samples; these two subtypes were distinguished by consensus clustering (Figs. **9A**-**C**). Fig. (**9D**) presents a heatmap, created using the pheatmap tool, illustrating the differences in expression of these 12 module genes between the two subtypes. The significant differences between the subtypes were further validated by PCA clustering (Fig. **9E**).

To validate these findings, a gene comparison chart (Fig. **9F**) revealed that nine module genes (FGF2, IL-11, IL-18, IL-1RN, IL-27, IL-4, NOG, TNF-α, TNF-β) exhibited significantly different expression levels between the two pancreatic cancer subtypes.

### 3.9 Immunotherapy and Immune Infiltration Analysis

**Fig. (10):**
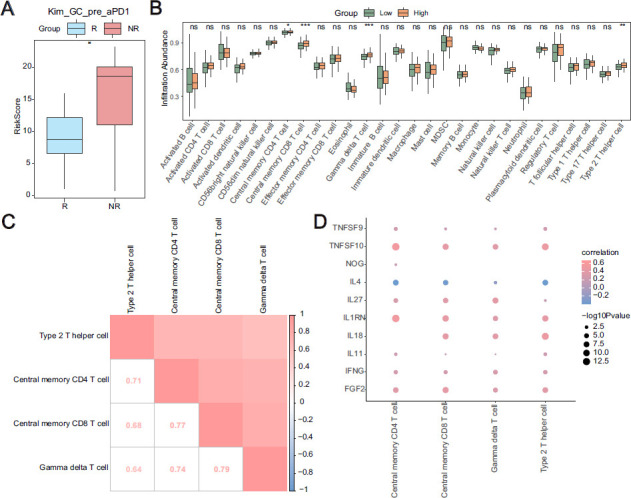
Immune infiltration analysis of TCGA-GTEx-PAAD dataset. (**A**) Kim (2018) used the Gastric Cancer (GC) immunotherapy dataset to create a group comparison plot of differential efficacy. (**B**) The TCGA-GTEx-PAAD dataset shows a clustering comparison plot of immune cells in two groups: high and low. The samples include pancreatic cancer. (**C**) A heatmap showing the correlation between the TCGA-GTEx-PAAD dataset and the quantity of immune cell infiltration in pancreatic cancer samples. (**D**) The quantity of immune cells infiltrating TCGA-GTEx-PAAD pancreatic cancer samples and the correlation with module genes is shown in the dot plot. PAAD refers to pancreatic adenocarcinoma, and TCGA refers to The Cancer Genome Atlas. The absolute value of the correlation coefficient (R-value) less than 0.3 indicates weak or no association. A weak correlation is defined as 0.3 to 0.5, while a moderate correlation is defined as 0.5 to 0.8. Positive correlations are represented by red, while negative correlations are indicated by blue. The color depth shows the intensity of the association. The R group is represented in blue, the NR group in red, the low-risk group in green, and the high-risk group in orange. Statistical significance is denoted as ns (*p* ≥ 0.05, no significance), * (*p* < 0.05), ** (*p* < 0.01), *** (*p* < 0.001).

To evaluate differences in immunotherapy efficacy between the high-risk and low-risk cohorts within the pancreatic cancer prognostic framework, RiskScores from the GC immunotherapy dataset (Kim 2018) were analyzed. Patients achieving a complete or partial response (R group) displayed significantly lower RiskScores, indicating that low-risk individuals were more likely to benefit from immunotherapy (Fig. **10A**).

In the TCGA-GTEx-PAAD study, the percentage of 28 immune cell types was measured using the ssGSEA method. Notable variations in immune cell infiltration were observed between the high- and low-risk groups for four specific cell types: CD4+ T cells with central memory, CD8+ T cells with central memory, gamma delta T cells, and type 2 T helper cells (*p* < 0.05, Fig. **10B**). Positive correlations between these immune cell types were displayed in a heatmap (Fig. **10C**).

A correlation dot plot (Fig. **10D**) revealed a significant negative correlation between the module gene *IL4* and central memory CD4+ T cells (R = -0.39, *p* < 0.001), while *TNFSF10* showed a positive correlation with central memory CD4+ T cells (R = 0.53, *p* < 0.001).

### 3.10 Experimental Validation of Key Prognostic Genes

To validate bioinformatics predictions and confirm the clinical relevance of the identified biomarkers, quantitative real-time PCR analysis was performed on selected genes from our prognostic model using paired PAAD tumor tissues and adjacent normal tissues from 20 patients with pancreatic cancer.

**Fig. (11):**
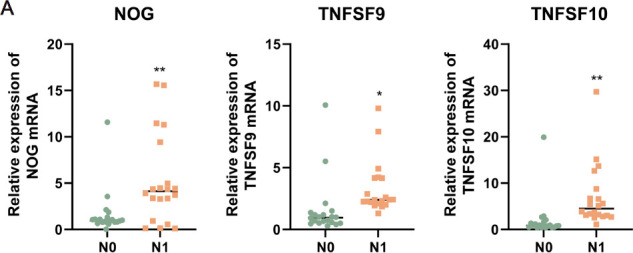
PCR Validation of differentially expressed genes in PAAD samples. (**A**) Quantitative real-time PCR analysis showing relative expression levels of NOG, TNFSF9, and TNFSF10 in adjacent normal tissues (N0) *versus* tumor tissues (N1). Data points represent individual paired samples with mean ± SEM. Statistical significance is indicated as ns (*p* ≥ 0.05), * (*p* < 0.05), ** (*p* < 0.01), *** (*p* < 0.001).

The experimental validation revealed significant differential expression patterns consistent with our computational analysis. Three key genes showed notable expression differences between adjacent normal tissues (N0) and tumor tissues (N1) (Fig. **11**). NOG exhibited significantly higher expression in tumor tissues compared to adjacent normal tissues (*p* < 0.01), confirming its upregulation in PAAD and its potential oncogenic role. TNFSF9 showed moderate but significant upregulation in tumor samples (*p* < 0.05), while TNFSF10 displayed a highly significant increase in expression in tumor tissues (*p* < 0.01).

Notably, the upregulation of TNFSF10 in tumor samples corroborates our earlier finding of its positive correlation with central memory CD4+ T cells, suggesting that increased TNFSF10 expression in the TME may contribute to immune dysfunction and tumor progression.

These PCR validation results not only confirm the reliability of our computational approach but also provide experimental evidence for the clinical significance of these immune- and mitochondria-related genes in PAAD tumorigenesis, supporting their potential as therapeutic targets and prognostic biomarkers.

## 4 DISCUSSION

This study introduces an innovative “Immune Dictionary” framework to systematically analyze immune-related genes (DIRRGs) and MRGs within the TME of PAAD. By integrating these two critical aspects of tumor biology, DEGs associated with the aggressive behavior of PAAD were identified, highlighting the significance of immune suppression and mitochondrial dysfunction in PAAD progression.

The immune microenvironment plays a pivotal role in the progression of PAAD. Among the hub genes identified in our analysis, *EGF* promotes pancreatic cancer cell proliferation by activating the epidermal growth factor receptor (EGFR), which in turn drives signaling pathways such as MAPK and p38 MAPK [49]. FGF2 enhances mitogenic activity through binding to Glypican-1, promoting cell growth, and inducing pancreatic ductal hyperplasia and stromal fibrosis *in vivo* [50].

The interleukin network plays a central role in PAAD immune evasion. GSVA revealed key pathways enriched in high-risk patients with PAAD, particularly IL-4 signaling [51, 52]. IL11 activates pancreatic stellate cells to form physical barriers that hinder immune cell infiltration, while IL13 and IL4 promote Th2-type immune responses and M2 macrophage polarization. IL18, with dual roles, may contribute to immunosuppression *via* regulatory T-cell (Treg) expansion. IL27, despite its anti-tumor properties, may facilitate immune evasion in advanced PAAD by upregulating PD-L1. Recent studies have shown that IL-4 signaling not only promotes M2 macrophage polarization but also directly modulates the metabolic landscape of tumor cells, creating a feed-forward loop that sustains immunosuppression [53].

Although IFNG possesses anti-tumor properties, its chronic exposure can lead to adaptive resistance mechanisms through increased PD-L1 expression. TNFSF10 (TRAIL), a hub gene, exhibits selective cytotoxicity; however, PAAD cells often develop TRAIL resistance through the upregulation of decoy receptors. NOG promotes epithelial-mesenchymal transition and stem cell-like properties by antagonizing BMP signaling.

NF-κB activation is linked to Treg recruitment, which suppresses the cytotoxic activity of CD^8+^ T cells and NK cells, thereby enhancing immune evasion [54, 55]. Recent research suggests that NF-κB signaling coordinates mitochondrial biogenesis with immune evasion mechanisms, revealing a direct connection between metabolic reprogramming and immunosuppression in PAAD [56]. Autophagy plays a dual role in cancer, enabling tumor cell survival under stress while contributing to therapy resistance and immune evasion [57-59]. Contemporary studies have shown that selective autophagy of mitochondria (mitophagy) is crucial in PAAD, as it allows cancer cells to eliminate damaged mitochondria while maintaining adequate ATP production to support rapid proliferation and metastasis.

The prognostic model developed based on 12 DEGs related to DIRRGs and MRGs effectively stratified patients into high- and low-risk groups. High-risk patients exhibited significantly poorer survival outcomes, underscoring the value of integrating immune and metabolic markers for PAAD risk assessment [60-64]. Immune infiltration analysis revealed that high-risk patients had lower levels of CD8+ T cells, NK cells, and gamma delta T cells, while showing increased levels of Tregs, which are known to promote tumor progression [65-67].

A key finding of this study is the significant correlation between immune evasion and mitochondrial dysfunction in patients with PAAD. Spearman's correlation analysis showed significant associations between MRGs and immune cell infiltration, particularly genes involved in oxidative phosphorylation (OXPHOS), which correlated positively with CD8+ T cells and NK cells. These results align with recent mechanistic studies demonstrating that OXPHOS capacity directly influences T-cell effector function and memory formation in cancer contexts [68-70].

Mitochondrial dysfunction, particularly prominent in high-risk patients, likely contributes to immune cell exhaustion, thereby reducing the efficacy of tumor elimination. Recent studies have identified specific mitochondrial metabolites and signaling pathways that regulate immune cell exhaustion, offering novel targets for therapeutic intervention [71-73]. Therapeutic strategies targeting mitochondrial function, particularly OXPHOS, may improve the efficacy of PAAD treatment. Current clinical trials investigating mitochondria-targeted therapies in combination with immune checkpoint inhibitors have shown promising preliminary results, supporting our computational predictions [74-77].

Our findings suggest that these genes form a complex regulatory network driving tumor progression and immune evasion, offering potential targets for combination immunotherapy. Targeting IL-4 signaling, NF-κB activation, and autophagy may enhance treatment efficacy in PAAD. Importantly, several identified genes exhibit stable expression patterns and are detectable in clinical specimens, highlighting their potential as diagnostic biomarkers or therapeutic response indicators in personalized treatment strategies.

This study provides novel insights into the relationship between mitochondrial dysfunction and the immune microenvironment in PAAD through integrative bioinformatic analyses. PCR validation of key genes (NOG, TNFSF9, TNFSF10) in clinical samples provides experimental support for our computational findings. However, the reliance on public datasets and computational methods is a key limitation. Dataset heterogeneity, including variations in sample origin and clinical background, may influence statistical outcomes, though consistent gene expression trends across cohorts support their biological relevance.

Future research should incorporate multi-cohort validation with larger sample sizes to address clinical variability and enhance robustness. Based on our PCR validation results, subsequent studies will further explore the functional mechanisms of these key genes. Moreover, combining mitochondria-targeted strategies with immune checkpoint inhibitors holds promise for overcoming immune evasion in PAAD. Targeting key hub genes and pathways identified in this study, particularly within personalized treatment frameworks, warrants further investigation.

## CONCLUSION

This study established a robust 12-gene prognostic signature by integrating immune-related and mitochondria-related genes in PAAD through an innovative “Immune Dictionary” framework. The findings highlight critical immune–mitochondrial crosstalk, with hub genes such as EGF and FGF2, as well as key interleukin networks, orchestrating immunosuppressive processes. The model effectively stratifies patients into risk groups with significant survival differences and identifies therapeutic targets, including IL-4 signaling, NF-κB activation, and autophagy, as candidates for combination immunotherapy. These biomarkers provide promising avenues for developing personalized treatment strategies in pancreatic cancer.

## Figures and Tables

**Table 1:** PAAD data set information list.

-	**TCGA-GTEx-PAAD**	**GSE15471**	**GSE28735**	**GSE62452**
Platform	/	GPL570	GPL6244	GPL6244
Species	Homo sapiens	Homo sapiens	Homo sapiens	Homo sapiens
Samples in the PAAD group	178	39	45	69
Samples in the Control group	332	39	45	61
Reference	/	Combined gene expression analysis of whole-tissue and micro-dissected pancreatic adenocarcinoma identifies genes specifically overexpressed in tumor epithelia.	DPEP1 inhibits tumor cell invasiveness, enhances chemosensitivity, and predicts clinical outcome in pancreatic adenocarcinoma	A novel MIF signaling pathway drives the malignant character of pancreatic cancer by targeting NR3C2.

**Abbreviations:** TCGA, The Cancer Genome Atlas; PAAD, Pancreatic Adenocarcinoma; GEO, Gene Expression Omnibus.

**Table 2:** GSEA enrichment analysis results.

**Description**	**SetSize**	**NES**	***p* Adjust**	**q Values**
REACTOME_AUTOPHAGY	149	1.4681	0.038528	0.036337
REACTOME_MITOPHAGY	28	1.89301	0.020488	0.019323
REACTOME_FCERI_MEDIATED_NF_KB_ACTIVATION	136	2.058266	1.39 e-08	1.31 e-08
PID_IL4_2PATHWAY	64	1.641212	0.04289	0.040451

**Abbreviation:** GSEA, Gene Set Enrichment Analysis.

**Table 3:** GO/KEGG enrichment analysis results.

Ontology	ID	Description	GeneRatio	BgRatio	*p* Adjust	q Value
BP	GO:1903578	Regulation of ATP metabolic process	2/12	89/18800	0.006651	0.001945
BP	GO:0051052	Regulation of DNA metabolic process	3/12	472/18800	0.010969	0.003207
BP	GO:0035265	Organ growth	2/12	175/18800	0.018234	0.005332
BP	GO:0050864	Regulation of B-cell activation	2/12	200/18800	0.021369	0.006248
BP	GO:0051132	NK T cell activation	1/12	13/18800	0.023758	0.006947
MF	GO:0005125	Cytokine activity	10/12	235/18410	1.96 e-16	9.02 e-17
MF	GO:0048018	Receptor ligand activity	11/12	489/18410	6.08 e-16	2.8 e-16
MF	GO:0030546	Signaling receptor activator activity	11/12	496/18410	6.08 e-16	2.8 e-16
MF	GO:0008083	Growth factor activity	4/12	162/18410	1.44 e-05	6.65 e-06
MF	GO:0005149	Interleukin-1 receptor binding	1/12	17/18410	0.03529	0.016252
KEGG	hsa04630	JAK-STAT signaling pathway	5/12	162/8164	5.12 e-05	2.66 e-05
KEGG	hsa04657	IL-17 signaling pathway	3/12	94/8164	0.003771	0.001958
KEGG	hsa04660	T cell receptor signaling pathway	2/12	104/8164	0.03486	0.018103
KEGG	hsa04066	HIF-1 signaling pathway	2/12	109/8164	0.036419	0.018913
KEGG	hsa04151	PI3K-Akt signaling pathway	3/12	354/8164	0.043301	0.022486

**Abbreviations:** GO, Gene Ontology; KEGG, Kyoto Encyclopedia of Genes and Genomes; BP, Biological Process; MF, Molecular Function.

**Table 4:** GSVA enrichment analysis results.

Description	logFC	*p* adjust
HALLMARK_INTERFERON_ALPHA_RESPONSE	0.33091	1.31 e-06
HALLMARK_INTERFERON_GAMMA_RESPONSE	0.28073	2.42 e-06
HALLMARK_TGF_BETA_SIGNALING	0.28033	1.31 e-06
HALLMARK_EPITHELIAL_MESENCHYMAL_TRANSITION	0.25053	0.000166
HALLMARK_NOTCH_SIGNALING	0.23537	1.07 e-05
HALLMARK_APICAL_SURFACE	0.22317	3.10 e-06
HALLMARK_GLYCOLYSIS	0.21021	2.32 e-05
HALLMARK_HYPOXIA	0.20657	2.21 e-05
HALLMARK_MITOTIC_SPINDLE	0.20326	8.18 e-05
HALLMARK_APICAL_JUNCTION	0.19773	3.02 e-05
HALLMARK_COMPLEMENT	0.14186	0.004107
HALLMARK_UV_RESPONSE_DN	0.13992	0.008738
HALLMARK_IL6_JAK_STAT3_SIGNALING	0.13906	0.014687
HALLMARK_HEDGEHOG_SIGNALING	0.13824	0.009748
HALLMARK_PI3K_AKT_MTOR_SIGNALING	0.13085	0.004287
HALLMARK_KRAS_SIGNALING_UP	0.12702	0.008738
HALLMARK_UV_RESPONSE_UP	0.12373	0.004287
HALLMARK_IL2_STAT5_SIGNALING	0.10635	0.026648
HALLMARK_WNT_BETA_CATENIN_SIGNALING	0.10422	0.040487
HALLMARK_HEME_METABOLISM	0.08319	0.044463

**Abbreviation:** GSVA, Gene Set Variation Analysis.

## Data Availability

The data analyzed in this study were obtained from publicly available databases, including The Cancer Genome Atlas (TCGA) (https://portal.gdc.cancer.gov/, TCGA-PAAD) and the Gene Expression Omnibus (GEO). https://www.ncbi. nlm.nih.gov/geo/.

## LIST OF ABBREVIATIONS

PAAD: Pancreatic Adenocarcinoma
TME: Tumor Microenvironment
DEGs: Differentially Expressed Genes
NK: Natural Killer
EGFR: Epidermal Growth Factor Receptor
CDF: Cumulative Distribution Function
TCGA: The Cancer Genome Atlas

## STANDARDS FOR REPORTING

STROBE guidelines and methodologies were followed.
