# Supplementary material for: Immune-Mitochondrial Crosstalk in Pancreatic Adenocarcinoma: Systematic Identification of Prognostic Biomarkers Through Immune Dictionary Framework
Source: Endocr Metab Immune Disord Drug Targets. 2025 Oct 15;26:E18715303422686. doi: 10.2174/0118715303422686250925054821 (PMC13284654; doi:10.2174/0118715303422686250925054821)
Supplement: Supplementary file 1 [file EMIDDT-26-E18715303422686_SD1.pdf]

Supplementary Material

Immune-Mitochondrial Crosstalk in Pancreatic Adenocarcinoma: Systematic Identification of Prognostic Biomarkers Through Immune Dictionary Framework

Jiangang Zhao[1,2,#], Fenglin Zhang[3,4,#], Xinghe Liao[3,4], Ke Zhang[5,6], Ping Li[3,4,*] and Hao Chen[5,6,*]

[1]Department of Oncology, Shaoxing Central Hospital, Shaoxing, 312030, China; [2]Department of Oncology, The Central Affiliated Hospital, Shaoxing University, Shaoxing, 312030, China; [3]Oncology Department of Integrated Traditional Chinese and Western Medicine, The First Affiliated Hospital of Anhui Medical University, Hefei, 230022, China; [4]Graduate School of Anhui University of Traditional Chinese Medicine, Hefei, 230022, China; [5]Department of Integrative Oncology, Fudan University Shanghai Cancer Center, Shanghai 200032, China; [6]Department of Oncology, Shanghai Medical College, Fudan University, Shanghai 200032, China

Table S1.

DIR&MRDEGs	DIR&MRGs
TNF	TNF
TNFSF10	TNFSF10
IL6	IL6
IL1B	IL1B
FASLG	FASLG
TGFB1	TGFB1
LEP	IGF1
ADIPOQ	LEP
IFNG	ADIPOQ
IFNA1	IFNG
EGF	IFNA1
IFNB1	EGF
FGF2	IFNB1
THPO	FGF2
IL4	THPO
HGF	IL4
IL1A	HGF
TNFSF15	IL1A
RETN	TNFSF15
IL10	RETN
IL18	IL10
KITLG	IL18
C3	KITLG
IL2	C3
IL17A	IL2
IL33	IL17A
PRL	CSF3

GDNF	IL33
IL1RN	VEGFA
CD40LG	PRL
DCN	GDNF
IL13	IL1RN
IL9	CD40LG
CSF2	DCN
TNFSF11	IL24
CD70	IL3
C5	IL13
IL5	IL9
NOG	CSF2
TNFSF14	TNFSF11
IL7	CD70
IL11	C5
IL21	IL22
TSLP	IL5
TNFSF9	NOG
TNFSF12	IL15
TNFSF13B	TNFSF14
LIF	IL7
IL25	CSF1
IL27	IL11
OSM	IL21
CTF1	TSLP
	TNFSF9
	TNFSF12
	TNFSF13B
	LIF
	IL25
	IL27
	OSM
	CTF1
	IFNK
